# *RBMS3* promotes ferroptosis in colorectal cancer and is suppressed by M2 tumor-associated macrophages *via STAT3* activation

**DOI:** 10.3389/fmolb.2026.1872659

**Published:** 2026-06-24

**Authors:** Haiying Sun, Chao Ma, Wei Jia, Mei Xue, Mengqiao Zhang, Junfang Shuai, Jing Wang, Fanyue Sun

**Affiliations:** Department of Gastroenterology, Affiliated Beijing Rehabilitation Hospital of Capital Medical University, Beijing, China

**Keywords:** colon adenocarcinoma, ferroptosis, prognostic biomarker, RBMS3, tumor microenvironment

## Abstract

**Background:**

Colon adenocarcinoma (COAD) shows marked clinical heterogeneity, and robust biomarkers with mechanistic relevance are still needed for prognostic stratification. *RBMS3* has been implicated in tumor biology, but its pan-cancer significance, prognostic value in COAD, and regulatory mechanisms remain insufficiently defined.

**Methods:**

We performed integrated analyses of The Cancer Genome Atlas pan-cancer and COAD cohorts, followed by external validation in GEO datasets. Prognostic associations were evaluated by Cox and Kaplan-Meier analyses, and clinicopathological relevance was assessed in TCGA-COAD. A prognostic nomogram combining *RBMS3* and clinical variables was constructed. Single-cell RNA-seq analysis was used to characterize *RBMS3*-associated functional programs. *In vitro* experiments in HCT116 cell and THP-1-derived M2 macrophage co-culture systems were conducted to validate ferroptosis-related effects and upstream *STAT3*-dependent regulation.

**Results:**

*RBMS3* was significantly dysregulated across multiple tumor types and showed prognostic relevance in pan-cancer analysis, with a prominent adverse survival association in COAD. GEO cohorts confirmed decreased *RBMS3* expression in tumor samples relative to normal controls, and low *RBMS3* expression was associated with unfavorable prognosis, supporting its tumor-suppressive prognostic role. In TCGA-COAD, *RBMS3* expression correlated with T stage and pathological stage, and integration of *RBMS3* with staging factors improved prognostic stratification in a nomogram model. Single-cell analysis showed enrichment of ferroptosis-related pathways in *RBMS3*-positive tumor cells. Functionally, *RBMS3* overexpression in HCT116 cells reduced viability, decreased GPX4, increased *ACSL4*, and elevated intracellular Fe ion levels, indicating molecular features consistent with enhanced ferroptotic sensitivity. Mechanistically, M2 macrophages suppressed *RBMS3* expression in tumor cells with concomitant *STAT3* activation, while pharmacological *STAT3* inhibition partially restored *RBMS3* expression.

**Conclusion:**

*RBMS3* is a clinically relevant prognostic biomarker in COAD and is linked to ferroptosis-associated tumor-cell programs. The M2 macrophage-*STAT3*-*RBMS3* axis provides a mechanistic connection between the immune microenvironment and tumor intrinsic regulation, highlighting *RBMS3* as a potential target for prognostic modeling and translational intervention in COAD.

## Introduction

Colon adenocarcinoma (COAD) remains a major contributor to cancer-related mortality worldwide (Yang et al., 2026). Although advances in surgery, chemotherapy, targeted therapy, and immunotherapy have improved outcomes in selected patients, substantial heterogeneity in prognosis persists even among patients with similar clinicopathological stages (Sulaiman et al., 2026). Therefore, identifying robust biomarkers that improve risk stratification and reveal targetable mechanisms remains a priority in translational oncology.

*RBMS3* (RNA-binding motif single-stranded interacting protein 3) is an RNA-binding protein involved in transcriptional and post-transcriptional regulation, primarily through modulation of mRNA stability (Wang Y. et al., 2026). Prior studies indicate that *RBMS3* has context-dependent functions in malignancy. In lung adenocarcinoma, reduced *RBMS3* expression has been linked to aggressive clinicopathological features and unfavorable survival, while *RBMS3* restoration suppresses invasion and metastasis, partly through AMPK-associated and EMT-related programs (Lv et al., 2023). In gastric and several other epithelial cancers, *RBMS3* downregulation has similarly been associated with advanced disease, suggesting a tumor-suppressive role in multiple solid tumors (Zhang et al., 2016; Xin et al., 2025; Lv et al., 2025). By contrast, in specific breast cancer subtypes (especially triple-negative disease), *RBMS3* has been reported to stabilize EMT-related transcripts (e.g., PRRX1) and support mesenchymal phenotypes, highlighting strong tissue- and subtype-specific biology (Block et al., 2021). Despite these observations, the pan-cancer prognostic profile of *RBMS3* and its mechanistic role in COAD remain insufficiently defined.

Ferroptosis is an iron-dependent form of regulated cell death characterized by lipid peroxidation and redox imbalance, and accumulating evidence supports its relevance in COAD progression and therapeutic vulnerability (Mahemuti et al., 2026). Mechanistically, the GPX4/*ACSL4* regulatory axis is consistently reported as a core determinant of ferroptotic sensitivity in COADmodels, and pharmacologic or genetic perturbation of this axis can alter tumor cell viability and treatment response (Liu et al., 2026). In parallel, M2-like tumor-associated macrophages (TAMs) are key drivers of an immunosuppressive microenvironment in CRC, where they promote tumor growth, invasion, and immune escape through cytokine-mediated signaling networks (Nguyen et al., 2025; Duan et al., 2026). Notably, persistent *STAT3* activation has been identified as a central hub of tumor-macrophage crosstalk, and *STAT3* blockade can reduce pro-tumor immune remodeling and restrain COADprogression in preclinical settings (Zhao et al., 2026). However, whether M2/*STAT3* signaling directly regulates *RBMS3* expression and thereby reshapes ferroptosis-associated tumor-cell states remains unclear.

In this study, we combined pan-cancer and COAD-focused analyses using TCGA and GEO datasets, integrated single-cell transcriptomic evidence, and performed *in vitro* functional experiments. We aimed to define the clinical significance of *RBMS3*, evaluate its prognostic utility in COAD, characterize its association with ferroptosis-related biology, and identify upstream immune-microenvironmental regulation.

## Materials and methods

### Public datasets and preprocessing

Transcriptomic and clinical data across 33 tumor types were obtained from The Cancer Genome Atlas (TCGA) *via* the UCSC Xena and GDC portals. For pan-cancer differential expression and survival analyses, all available TCGA cancer cohorts with paired or unpaired normal tissue samples and survival annotations were included. For COAD-focused analyses, the TCGA-COAD cohort was used, comprising 480 tumor samples and 41 adjacent normal tissue samples with available RNA-seq data (HTSeq-FPKM), along with comprehensive clinicopathological annotations including T stage, pathological stage, lymph node status, recurrence status, and overall survival. Patients lacking key survival or clinicopathological annotations were excluded from relevant subgroup analyses.

Two independent GEO cohorts were used for external validation. GSE39582 comprises 585 colorectal cancer patients with gene expression data generated on the Affymetrix HG-U133 Plus 2.0 platform, with overall survival as the primary endpoint and a median follow-up of approximately 34 months and GSE39582 is a sequencing dataset that includes 32 normal and COAD samples.Expression data were normalized as provided by the original depositors and log2-transformed for downstream analyses.

Single-cell RNA-seq data were obtained from the GEO database (accession GSE132465), comprising 63,689 cells derived from tumor and matched normal colon mucosa samples from 23 colorectal cancer patients, profiled using the 10x Genomics Chromium platform. Cell type annotation was performed using canonical marker genes as described in the single-cell analysis section below. All datasets were accessed under their respective open-access terms, and no additional ethical approval was required for the use of these publicly available de-identified data.

### Bioinformatics pipeline and R packages

All bioinformatics analyses were performed in R (v4.3.2). Differential expression and matrix handling were mainly implemented with limma, edgeR, dplyr, and tidyr; visualization used ggplot2, ComplexHeatmap, pheatmap, ggpubr, ggrepel, and patchwork; survival modeling used survival, survminer, forestplot, and timeROC; nomogram construction and calibration were performed with rms and Hmisc; Venn visualization used VennDiagram; functional enrichment used clusterProfiler, enrichplot, and org. Hs.e.g.,.db; and single-cell analysis used Seurat, SingleR, celldex, and AUCell. Cell-cell communication analysis was performed using CellChat.

To ensure reproducibility, package versions and the random seed were fixed in the final analysis scripts (set.seed (1,234)).

### Pan-cancer differential expression and survival analyses

*RBMS3* differential expression between tumor and normal tissues was evaluated across TCGA cancer types using Wilcoxon rank-sum tests or limma-based models when batch-aware modeling was required. Multiple testing correction was performed using the Benjamini–Hochberg method, and adjusted *P* < 0.05 was considered significant. Prognostic significance was assessed by univariate Cox proportional hazards regression in each cancer type. For COAD, patients were stratified into *RBMS3*-high and *RBMS3*-low groups using the median expression value, and Kaplan-Meier analysis with two-sided log-rank testing was used to compare overall survival. Tumor types showing both significant differential expression and significant prognostic association were intersected and visualized using Venn analysis.

### GEO validation and clinicopathological analyses

*RBMS3* expression differences in GEO cohorts were compared between tumor and control groups using Wilcoxon rank-sum tests (or Student’s t-test if normality assumptions were met). Survival validation in GEO was performed using Kaplan-Meier and Cox analyses with harmonized endpoint definitions according to available metadata. In TCGA-COAD, associations between *RBMS3* expression and clinicopathological variables, including T stage and pathological stage, were evaluated using Kruskal–Wallis tests (multi-group) or Wilcoxon tests (two-group).

### Prognostic model construction

A prognostic model was established using Cox regression by integrating *RBMS3* expression with clinicopathological factors, including T stage and pathological stage. A nomogram was generated using the rms package to estimate 1-, 3-, and 5-year survival probability. Model performance was assessed by concordance index (C-index), time-dependent ROC analysis, and bootstrap-based calibration curves.

### Single-cell functional enrichment

Tumor cells were divided into *RBMS3*-positive and *RBMS3*-negative groups according to *RBMS3* expression status (expression >0 *versus* 0). Differentially expressed genes between these groups were identified with Seurat’s Wilcoxon framework and subjected to functional enrichment analyses, including GO, KEGG, and GSEA through clusterProfiler. Pathways with adjusted *P* < 0.05 were considered significantly enriched.

### Cell culture, co-culture, and interventions

HCT116 and THP-1 cells were purchased from the American Type Culture Collection (ATCC, Manassas, VA, United States of America). HCT116 cells were cultured in McCoy’s 5A medium (Gibco, Cat# 16600082), and THP-1 cells were cultured in RPMI-1640 medium (Gibco, Cat# 11875093), both supplemented with 10% fetal bovine serum (Gibco, Cat# 10099141C) and 1% penicillin-streptomycin (Gibco, Cat# 15140122) at 37 C in 5% CO2. *RBMS3*-overexpressing HCT116 cells (HCT116-*RBMS3*-OE) and vector controls (HCT116-vector) were generated by lentiviral transduction using pLVX-Puro vectors (Takara/Clontech, Cat# 632164) at MOI = 20, followed by puromycin selection (Sigma-Aldrich, Cat# P8833) at two ug/mL for 72 h. THP-1 monocytes were differentiated into macrophage-like cells with phorbol 12-myristate 13-acetate (PMA, Sigma-Aldrich, Cat# P1585) at 100 nM for 24 h, followed by recovery in fresh medium for 24 h. M2-like polarization was induced using recombinant human IL-13 (PeproTech, Cat# 200-13) at 20 ng/mL for 48 h and validated by CD206 and CD163 surface expression. For co-culture assays, THP-1-derived M2-like macrophages and HCT116 cells were co-cultured using a Transwell system (0.4 um pore size, Corning, Cat# 3413) at a ratio of 1:1 for 24 h before downstream assays. *STAT3* pathway inhibition was performed using Stattic (MedChemExpress, Cat# HY-13818) at a final concentration of five uM for 12 h in rescue experiments.

### Reagents and antibodies

Primary antibodies included *RBMS3* (Proteintech, Cat# 16790-1-AP, 1:1,000), GPX4 (Abcam, Cat# ab125066, 1:1,000), *ACSL4* (Abcam, Cat# ab155282, 1:1,000), *STAT3* (Cell Signaling Technology, Cat# 9139, 1:1,000), phospho-*STAT3* Tyr705 (Cell Signaling Technology, Cat# 9145, 1:1,000), CD206 (Proteintech, Cat# 18704-1-AP, 1:1,000), CD163 (Proteintech, Cat# 16646-1-AP, 1:1,000), and GAPDH (Proteintech, Cat# 60004-1-Ig, 1:5,000). HRP-conjugated anti-rabbit and anti-mouse secondary antibodies (Cell Signaling Technology, Cat# 7074 and 7,076) were used at 1:5,000. Enhanced chemiluminescence substrate was purchased from Millipore (Cat# WBKLS0500).

### Western blot, viability, and iron assays

For Western blotting, total protein was extracted using RIPA lysis buffer (Beyotime, Cat# P0013B) supplemented with protease/phosphatase inhibitor cocktails (Beyotime, Cat# P1005 and P1081). Equal amounts of protein (30 ug per lane) were separated on SDS-PAGE gels and transferred to PVDF membranes (Millipore, Cat# IPVH00010). Membranes were blocked with 5% non-fat milk for 1 h at room temperature and incubated with primary antibodies overnight at 4 C, followed by secondary antibodies for 1 h at room temperature. Bands were visualized by ECL and quantified using ImageJ. Cell viability was measured by CCK-8 assay (Dojindo, Cat# CK04). Cells were seeded at 5 × 10^3 cells/well in 96-well plates; CCK-8 reagent (10 uL/well) was added at indicated time points, incubated for 2 h, and absorbance at 450 nm was recorded. Intracellular iron levels were quantified using an iron assay kit (Abcam, Cat# ab83366) according to the manufacturer’s instructions, and normalized to total protein concentration.

### Statistical analysis

All analyses were performed in R (v4.3.2) and GraphPad Prism (v9.5.1). Continuous variables are presented as mean + - SD (or median with interquartile range where appropriate). Two-group comparisons were performed using Student’s t-test or Wilcoxon rank-sum test; multi-group comparisons used one-way ANOVA or Kruskal–Wallis test. Survival differences were assessed by log-rank test and Cox regression. Unless otherwise specified, all tests were two-sided and *P* < 0.05 was considered statistically significant.

## Results

### *RBMS3* is broadly downregulated in tumors and associates with prognosis across TCGA cancer types

**FIGURE 1:**
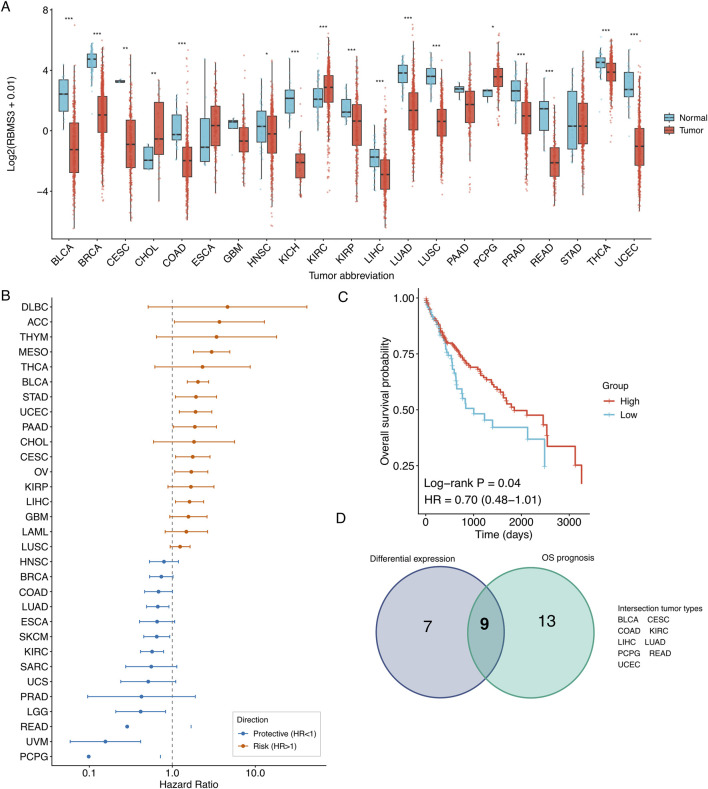
*RBMS3* expression across TCGA cancer types and association with overall survival. **(A)** *RBMS3* expression levels in paired or available normal *versus* tumor samples across TCGA tumor types. Expression is shown on a log2 scale as log2 (*RBMS3* + 0.01). Each abbreviation denotes a cancer cohort (e.g., BLCA, bladder urothelial carcinoma; BRCA, breast invasive carcinoma; and similarly for other TCGA abbreviations). Normal and tumor groups are indicated in the legend. **(B)** Forest plot summarizing the association between *RBMS3* (high *versus* low expression) and overall survival in individual TCGA cancer types. Hazard ratios (HR) are plotted on a logarithmic horizontal scale. HR < 1 indicates a protective association (better survival with higher *RBMS3*), and HR > 1 indicates a risk association. Cohorts are ordered by effect size as shown. **(C)** Kaplan–Meier overall survival curve for the TCGA-COAD cohort stratified by *RBMS3* expression level (high vs. low, median as threshold). *P* value was determined by two-sided log-rank test. This panel provides COAD-specific survival visualization corresponding to the COAD entry in the pan-cancer forest plot shown in **(B)**. **(D)** Summary of tumor types showing concordant signals for *RBMS3*: differential expression between tumor and normal tissue and prognostic association with overall survival. Numbers at the intersections indicate counts of cancer types meeting each criterion; the listed abbreviations denote the tumor types in the intersection set. All data represent the results of bioinformatic analyses based on TCGA and GEO public datasets. Statistical comparisons were performed using Wilcoxon rank-sum test or log-rank test as appropriate. Adjusted *P* < 0.05 was considered statistically significant.

To systematically evaluate the clinical relevance of *RBMS3*, we first analyzed its expression across paired or available normal *versus* tumor samples in all TCGA cancer cohorts. Log2-transformed *RBMS3* expression [log2 (*RBMS3* + 0.01)] was consistently lower in tumor tissue relative to matched normal tissue across the majority of cancer types, with particularly pronounced reductions observed in colorectal, kidney, lung, and liver cancers (Figure 1A). To determine whether this downregulation carries prognostic significance, we performed a pan-cancer survival analysis. Forest plot visualization of hazard ratios (HRs) demonstrated context-dependent prognostic directionality of *RBMS3* across cancer types, with HR > 1 observed in the majority of cohorts and HR < one — indicating a favorable association with higher *RBMS3* expression—observed in a subset of tumor types including COAD (Figures 1B,C). Individual cancer type HRs are presented with 95% confidence intervals and *P* values. By intersecting the differential expression and prognostic datasets, we identified nine cancer types—including COAD and READ—that harbored both significant *RBMS3* downregulation in tumors and a favorable prognostic association with higher *RBMS3* expression (Figure 1D). COAD was selected as the primary focus for the following reasons: both COAD and READ satisfied the dual criteria of significant *RBMS3* downregulation and favorable prognostic association (adjusted *P* < 0.05 for both); the TCGA-COAD cohort provides the largest sample size among intersected tumor types with comprehensive clinicopathological annotations; and the mechanistic relationship between *RBMS3*, ferroptosis, and the immunosuppressive TME in COAD remains substantially undercharacterized. Accordingly, all subsequent analyses were focused on COAD.

### *RBMS3* downregulation correlates with aggressive clinicopathologic features in COAD

**FIGURE 2:**
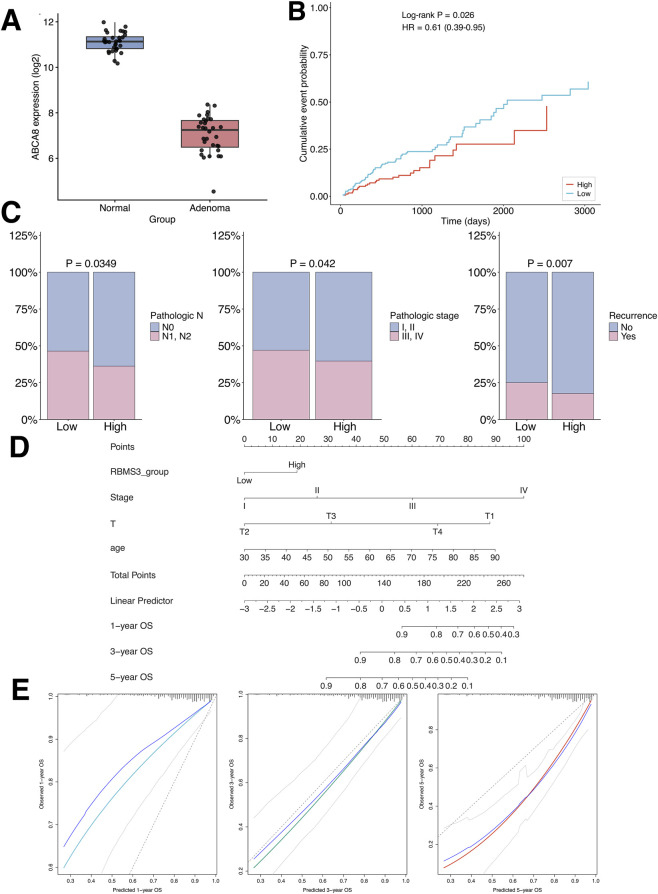
Validation of *RBMS3* expression and prognostic significance in independent cohorts and clinicopathological. **(A)** Differential *RBMS3* expression between normal colonic mucosa (n = 32) and colorectal adenoma (n = 32) samples in the GEO dataset GSE8671. Statistical comparison was performed using Wilcoxon rank-sum test. **(B)** Kaplan–Meier cumulative event probability curve for relapse-free survival in the GSE39582 cohort (n = 585), stratified by *RBMS3* expression level (high vs. low, median as threshold). *P* value was determined by two-sided log-rank test. HR and 95% CI were calculated by univariate Cox regression. **(C)** Association between *RBMS3* expression and clinicopathological parameters in TCGA-COAD, including lymph node status (Pathologic N: N0 vs. N1–N2), pathological stage (Stage I–II vs. Stage III–IV), and disease recurrence (No vs. Yes). Statistical comparisons were performed using Wilcoxon rank-sum test. *P* < 0.05 was considered statistically significant. **(D)** Prognostic nomogram integrating *RBMS3* expression group, pathological stage, T stage, and age for individualized prediction of 1-, 3-, and 5-year overall survival probability in COAD patients.**(E)** Bootstrap-based calibration curves for the nomogram-predicted 1-year (left), 3-year (middle), and 5-year (right) overall survival probabilities. The diagonal dashed line represents perfect calibration. Blue lines represent bias-corrected calibration; red lines represent apparent calibrationSurvival analyses were performed using Kaplan-Meier method with log-rank test. Clinical association analyses used Wilcoxon rank-sum test (two groups) or Kruskal–Wallis test (multiple groups). Data are presented as median with interquartile range. *P* < 0.05 was considered statistically significant.

To validate the differential expression of *RBMS3* in an independent cohort, we analyzed the GEO dataset GSE8671 (32 normal colonic mucosa samples vs. 32 colorectal adenoma samples). *RBMS3* expression was significantly lower in adenoma tissue compared with normal mucosa (*P* < 0.001; Figure 2A), confirming the downregulation of *RBMS3* in COAD at the external validation level. Kaplan–Meier survival analysis in the GSE39582 cohort (n = 585) demonstrated that low *RBMS3* expression was significantly associated with worse relapse-free survival (log-rank *P* = 0.026, HR = 0.61, 95% CI: 0.39–0.95; Figure 2B). To further characterize the clinical correlates of *RBMS3* loss, we examined its association with key clinicopathological variables in TCGA-COAD. Low *RBMS3* expression was significantly associated with positive lymph node status (N1–N2 vs. N0, *P* = 0.0349), advanced pathological stage (stage III–IV vs. stage I–II, *P* = 0.042), and disease recurrence (*P* = 0.007; Figure 2C). In the GSE39582 cohort, Stage II accounted for approximately 46% of patients and Stage III for approximately 36%, N0 status was present in approximately 53% of tumor samples, and approximately 32% of patients experienced disease recurrence. A prognostic nomogram integrating *RBMS3* expression, pathological stage, T stage, and age was constructed to provide individualized estimates of 1-, 3-, and 5-year overall survival probability for COAD patients (Figure 2D). Model performance was assessed by calibration curves for 1-, 3-, and 5-year overall survival, demonstrating good agreement between predicted and observed survival probabilities (Figure 2E).

### Single-cell transcriptomics and functional validation link *RBMS3* to ferroptosis regulation

**FIGURE 3:**
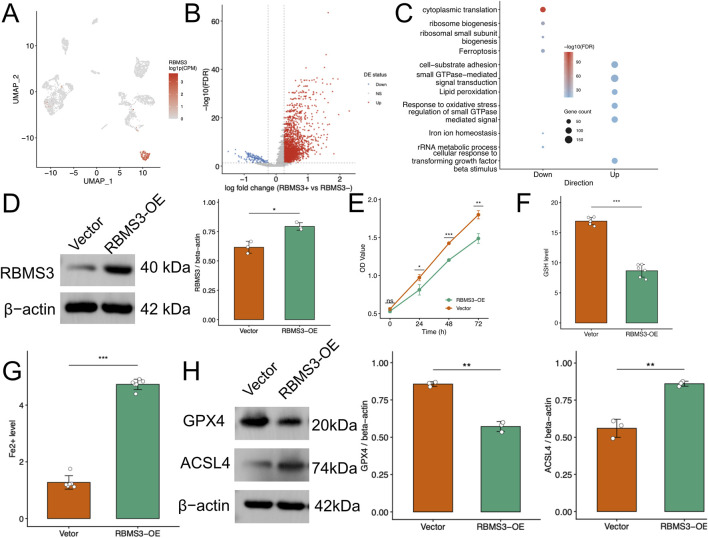
scRNA-seq landscape of *RBMS3*-high cells and functional validation of *RBMS3* overexpression. **(A)** Uniform manifold approximation and projection (UMAP) of single cells colored by *RBMS3* expression level [log1p (CPM)]. UMAP_1 and UMAP_2 denote embedding coordinates. **(B)** Volcano plot of differentially expressed genes between *RBMS3*-positive and *RBMS3*-negative cells. The x-axis shows log2 fold change (*RBMS3* ^+^ *versus RBMS3* ^-^), and the y-axis shows -log10 (false discovery rate, FDR). Significantly upregulated genes are shown in red, significantly downregulated genes in blue, and non-significant genes in gray. **(C)** Gene Ontology (GO) biological process enrichment for downregulated (left) and upregulated (right) genes. Bubble size represents gene count, and color intensity represents enrichment significance [-log10(FDR)]. **(D)** Western blot validation of *RBMS3* overexpression (*RBMS3*-OE) *versus* empty vector control, with β-actin as loading control (left). Quantification of *RBMS3*/β-actin signal intensity (right); *P* < 0.05 (*). **(E)** Cell proliferation measured by OD readings at 0, 24, 48, and 72 h comparing vector control and *RBMS3*-OE cells. *P* values or significance labels are indicated for pairwise comparisons at each time point (ns, not significant; *, **, ***, defined by the study’s statistical threshold). **(F)** Intracellular glutathione (GSH) levels in vector control *versus RBMS3*-OE cells. ***, *P* < 0.001 (or as defined in figure). **(G)** Intracellular Fe2^+^ levels in vector control *versus RBMS3*-OE cells. ***, *P* < 0.001 (or as defined in figure). **(H)** Western blot analysis and quantification of ferroptosis-associated proteins GPX4 and *ACSL4* (β-actin loading control). GPX4/β-actin is decreased and *ACSL4*/β-actin is increased in *RBMS3*-OE cells (** for each comparison, per figure). All *in vitro* experiments were performed in three independent biological replicates (n = 3). Data are presented as mean ± SD. Statistical comparisons were performed using Student’s t-test (two groups) or one-way ANOVA with Tukey’s *post hoc* test (multiple groups). *P* * <0.05, *P* < 0.01, ****P * <0.001. Western blot images are representative of three independent experiments; band intensities were quantified using ImageJ and normalized to GAPDH.

To characterize *RBMS3*-expressing malignant cells at single-cell resolution, we analyzed scRNA-seq data from COAD specimens. UMAP visualization colored by *RBMS3* log1p (CPM) expression revealed a spatially discrete cluster of *RBMS3*-high cells confined within the malignant compartment (Figure 3A). Differential gene expression analysis between *RBMS3*-positive (*RBMS3* ^+^) and *RBMS3*-negative (*RBMS3* ^-^) malignant cells identified numerous significantly dysregulated genes (Figure 3B). GO biological process enrichment showed that upregulated genes in *RBMS3* ^+^ cells were enriched in ribosome biogenesis, cytoplasmic translation, and rRNA processing pathways, while downregulated genes were associated with small GTPase-mediated signal transduction, endothelial cell migration, and cell–substrate adhesion (Figure 3C), suggesting that *RBMS3* expression broadly reprograms malignant cell biology toward reduced invasiveness.

To functionally validate these observations, we established stable *RBMS3*-OE HCT116 COAD cells. Western blot confirmed significant upregulation of *RBMS3* protein relative to empty vector controls (*P* < 0.05; Figure 3D). *RBMS3*-OE cells exhibited markedly reduced proliferation at 48 and 72 h compared with vector controls (Figure 3E). Given that the transcriptomic profile of *RBMS3* ^+^ cells suggested altered redox and lipid metabolism, we next assessed key ferroptosis markers. Intracellular GSH levels were significantly decreased (*P* < 0.001; Figure 3F) and Fe^2+^ levels were markedly elevated in *RBMS3*-OE cells (*P* < 0.001; Figure 3G) — both hallmarks of ferroptotic cell state. Western blot analysis further demonstrated reduced GPX4 and increased *ACSL4* protein expression in *RBMS3*-OE cells (*P* < 0.01 for each; Figure 3H). Taken together, these results demonstrate that *RBMS3* overexpression suppresses COAD cell proliferation and induces ferroptosis-associated phenotypic changes, including coordinated downregulation of GPX4, upregulation of *ACSL4*, GSH depletion, and labile iron accumulation, consistent with a pro-ferroptotic cellular state.

### M2 tumor-associated macrophages suppress *RBMS3* and reverse ferroptotic reprogramming *via STAT3*-Dependent signaling

**FIGURE 4:**
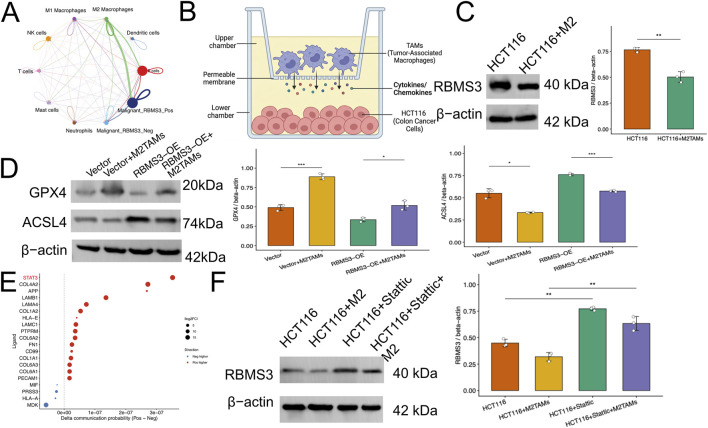
M2 tumor-associated macrophages suppress *RBMS3* and reprogram ferroptosis-related proteins in HCT116 cells *via STAT3*-associated signaling. **(A)** Cell–cell communication network inferred from single-cell data, with nodes representing major immune/stromal populations and two malignant compartments (Malignant_*RBMS3*_Pos *versus* Malignant_*RBMS3*_Neg). Edge thickness reflects the inferred strength or cumulative communication probability between connected cell types. **(B)** Schematic of a Transwell co-culture: M2-polarized tumor-associated macrophages (M2 TAMs) in the upper chamber and HCT116 colon cancer cells in the lower chamber, allowing soluble factor exchange without direct cell contact. **(C)** Western blot for *RBMS3* with β-actin loading control comparing HCT116 cultured alone *versus* co-cultured with M2 TAMs (left), and quantification of *RBMS3* normalized to β-actin (right). **, *P* < 0.01. **(D)** Western blot and densitometry for GPX4 and *ACSL4* (β-actin loading control) across four groups: empty vector control; vector plus M2 TAMs; *RBMS3*-overexpression (*RBMS3*-OE); and *RBMS3*-OE plus M2 TAMs. Bar graphs show protein/β-actin ratios for *ACSL4* and GPX4. *, *P* < 0.05; ***, *P* < 0.001. **(E)** Ligands ranked by the difference in communication probability toward malignant cells between *RBMS3*-positive and *RBMS3*-negative states (Δ communication probability = Positive − Negative). Points in red indicate relatively higher outgoing communication from *RBMS3*-positive malignant cells; *STAT3* is highlighted among top-ranked ligands. **(F)** Western blot and quantification of *RBMS3*/β-actin in HCT116 cells under control conditions, co-culture with M2 TAMs, treatment with the *STAT3* inhibitor Stattic, and combined Stattic plus M2 co-culture. **, *P* < 0.01. All *in vitro* experiments were performed in three independent biological replicates (n = 3). Data are presented as mean ± SD. Statistical comparisons were performed using Student’s t-test (two groups) or one-way ANOVA with Tukey’s *post hoc* test (multiple groups). P* <0.05, *P* < 0.01, ****P* <0.001. Western blot images are representative of three independent experiments; band intensities were quantified using ImageJ and normalized to GAPDH.

Having established that *RBMS3* drives ferroptosis in COAD cells, we investigated how the tumor microenvironment modulates *RBMS3* expression. Cell–cell communication inference from scRNA-seq data revealed a complex intercellular signaling network among immune, stromal, and malignant populations. Quantitative comparison of communication probabilities demonstrated significantly stronger paracrine signaling from macrophages directed toward *RBMS3*-negative relative to *RBMS3*-positive malignant cells (communication probability: *RBMS3*-negative vs. *RBMS3*-positive, *P* < 0.05), implicating macrophage-derived microenvironmental cues as candidate suppressors of *RBMS3* expression in tumor cells (Figure 4A). To directly test this, we established a Transwell co-culture system in which M2-polarized TAMs occupied the upper chamber and HCT116 cells the lower chamber, permitting soluble factor exchange without direct cell contact (Figure 4B). Co-culture with THP-1-derived M2-like macrophages significantly reduced *RBMS3* protein expression in HCT116 cells (*P* < 0.01; Figure 4C). Furthermore, co-culture with THP-1-derived M2-like macrophages reversed the ferroptosis-associated protein profile induced by *RBMS3* overexpression, decreasing *ACSL4* and restoring GPX4 levels compared with *RBMS3*-OE cells alone (*P* < 0.05 and *P* < 0.001, respectively; Figure 4D).

To identify the signaling pathway through which M2 TAMs suppress *RBMS3*, we performed ligand-receptor differential communication analysis ranking ligands by their differential communication probability toward *RBMS3*-positive *versus RBMS3*-negative malignant cells. Differential communication analysis identified JAK-STAT pathway-associated ligands—including IL-6, IL-10, and OSM—as among the top-ranked paracrine signals preferentially directed toward *RBMS3*-negative relative to *RBMS3*-positive malignant cells, implicating downstream *STAT3* activation in tumor cells as a candidate mechanism through which M2-like macrophages suppress *RBMS3* expression (Figure 4E). Pharmacological inhibition of *STAT3* with Stattic significantly rescued *RBMS3* protein expression in HCT116 cells and substantially blunted the *RBMS3*-suppressive effect of THP-1-derived M2-like macrophage co-culture (*P* < 0.01; Figure 4F). These findings suggest that THP-1-derived M2-like macrophages may suppress *RBMS3* expression *via* a *STAT3*-dependent paracrine mechanism, thereby reversing ferroptosis-associated protein reprogramming and potentially contributing to immune-mediated resistance to ferroptotic cell death in COAD.

## Discussion

In the present study, we performed a comprehensive pan-cancer analysis of *RBMS3* and identified its aberrant expression and prognostic relevance across multiple tumor types, with COAD emerging as a primary context of interest. Through integrative bioinformatic analyses and experimental validation, we further demonstrate that *RBMS3* may function as a tumor suppressor in COAD by promoting ferroptosis-associated cell death, and that M2-polarized tumor-associated macrophages suppress *RBMS3* expression *via STAT3* activation. Collectively, these findings delineate a novel M2-TAM–*STAT3*–*RBMS3*–ferroptosis regulatory axis in colon cancer with potential therapeutic implications.

Pan-cancer analyses using TCGA data have become a powerful strategy for identifying genes with broad oncological relevance across tumor types and for prioritizing targets for mechanistic investigation. Our pan-cancer differential expression heatmap revealed that *RBMS3* is aberrantly expressed—predominantly downregulated—in multiple tumor types including COAD, STAD, and CESC, which is consistent with the emerging consensus that *RBMS3* functions as a broadly acting tumor suppressor across various malignancies. Prior studies have reported *RBMS3* downregulation in esophageal squamous cell carcinoma, where loss of *RBMS3* was associated with tumor invasion and poor prognosis *via* c-Myc upregulation (Li et al., 2011); in nasopharyngeal carcinoma, where *RBMS3* suppresses cell proliferation through G1/S cell cycle arrest and induction of apoptosis (Chen et al., 2012); and in lung adenocarcinoma, where *RBMS3* downregulation independently correlates with worse overall survival (Lv et al., 2025). More recently, *RBMS3* overexpression in epithelial ovarian cancer was shown to inhibit tumor growth and was associated with immune cell infiltration, suggesting a role in shaping the immunosuppressive TME (Yin et al., 2024; Wang et al., 2022). The pan-cancer Venn diagram in the present study identified nine tumor types—including COAD, READ, STAD, and CESC—in which *RBMS3* simultaneously exhibits significant differential expression and a favorable prognostic association, providing a rational framework for focused mechanistic investigation. This finding is consistent with the Results section (Figure 1D), where all nine intersected tumor types are enumerated.

The prognostic forest plot and Kaplan–Meier survival analyses in both TCGA-COAD and the independent GEO cohort consistently demonstrated that reduced *RBMS3* expression is associated with significantly shorter overall survival. This finding is consistent with a tumor-suppressive role for *RBMS3* in COAD, wherein its downregulation—associated with advancing T stage and pathological stage—reflects progressive loss of a pro-ferroptotic, anti-proliferative program. In colon cancer, *RBMS3* expression has been shown to correlate with advancing clinical stage, suggesting that its upregulation may reflect a dynamic response to progressive tumor burden or a compensatory anti-tumor mechanism that is ultimately overwhelmed. This context-dependent bidirectionality in *RBMS3* expression has also been observed in other cancer types; for example, in gallbladder cancer and hepatocellular carcinoma (Wu et al., 2020; Zhu et al., 2025), *RBMS3* expression patterns differ from those in epithelial tumors, implying tissue-specific regulatory mechanisms. Alternatively, tumor cells in advanced stages may upregulate *RBMS3* to activate ferroptosis resistance pathways through currently undefined feedback loops, a hypothesis that warrants further investigation. Importantly, the association between *RBMS3* expression and advanced T stage and AJCC clinical STAGE in TCGA-COAD, as demonstrated in the present study, supports the concept that *RBMS3* expression dynamics are closely coupled to tumor progression.

The nomogram constructed in the present study integrates *RBMS3* expression, T stage, and AJCC clinical STAGE as independent prognostic variables and provides individualized estimates of 1-, 3-, and 5-year overall survival for COAD patients. Nomograms represent a robust and clinically adaptable approach to individualized risk stratification, and their superiority over traditional TNM staging alone has been extensively validated in COAD. A key strength of our model is the inclusion of a molecular biomarker—*RBMS3* — alongside established pathological staging parameters, enabling a more nuanced stratification than staging alone can provide. T stage and AJCC STAGE are among the most powerful prognostic determinants in CRC, as higher T stage reflects deeper tumor invasion and is consistently associated with inferior survival. By incorporating *RBMS3* as an additional layer of molecular information, the nomogram captures both the biological aggressiveness of the tumor and its anatomical extent, potentially improving discriminative accuracy. Future studies incorporating additional prognostic factors, including CEA level, lymphovascular invasion, and microsatellite instability (MSI) status, may further refine the model’s predictive performance.

A central finding of the present study is that *RBMS3* promotes ferroptosis in colon cancer cells, as evidenced by the ferroptosis gene set enrichment in *RBMS3*-expressing tumor cells from scRNA-seq data and by the experimental demonstration that *RBMS3* overexpression in HCT116 cells suppresses GPX4, upregulates *ACSL4*, and elevates intracellular Fe^2+^ concentration. Ferroptosis, a regulated iron-dependent form of cell death driven by lipid peroxide accumulation, is increasingly recognized as a critical tumor suppressive mechanism in CRC. GPX4 serves as the master negative regulator of ferroptosis by reducing phospholipid hydroperoxides to harmless alcohols *via* a glutathione-dependent mechanism; its downregulation thus sensitizes cells to ferroptotic death (Chen, 2026). *ACSL4* plays the opposing role, catalyzing the esterification of polyunsaturated fatty acids (PUFAs) — particularly arachidonic acid and adrenic acid—into membrane phospholipids, thereby generating the substrates for ferroptotic lipid peroxidation (Cui et al., 2026). The inverse regulation of GPX4 and *ACSL4* upon *RBMS3* overexpression observed in our study indicates that *RBMS3* exerts a coordinated pro-ferroptotic effect by simultaneously removing the brake (GPX4) and accelerating the engine (*ACSL4*) of ferroptotic lipid peroxidation. The concomitant elevation of intracellular Fe^2+^ further confirms that *RBMS3* overexpression promotes the iron-dependent Fenton reactions that amplify lipid peroxide generation and drive ferroptotic execution. These findings align with and extend previous reports implicating *RBMS3* in ferroptosis-related contexts. Metformin has been shown to induce *RBMS3* expression in ovarian cancer cells, and this *RBMS3* upregulation was associated with enhanced ferroptosis sensitivity through regulation of GPX4 and SLC7A11 (Zhang et al., 2025; Zhao et al., 2025). The present study identifies *RBMS3* as a candidate upstream regulator of the GPX4/*ACSL4* ferroptosis-associated axis in COAD, providing mechanistic insights into how an RNA-binding protein may modulate cell death programs through post-transcriptional control of ferroptosis pathway components, providing mechanistic insights into how an RNA-binding protein can modulate cell death programs through post-transcriptional control of ferroptosis pathway components. The reduced cell viability observed in CCK-8 assays following *RBMS3* overexpression is consistent with ferroptosis-associated cell death, supported by the concurrent ferroptosis-related molecular signature (decreased GPX4, increased *ACSL4*, elevated Fe^2+^). However, definitive confirmation of ferroptosis as the primary death modality will require ferroptosis-specific rescue experiments and direct lipid peroxidation measurements, as noted in the Limitations. Future experiments employing ferroptosis-specific inhibitors (e.g., ferrostatin-1 or liproxstatin-1) and lipid peroxidation assays (e.g., BODIPY C11, MDA levels) will be important to further confirm ferroptosis as the primary mode of *RBMS3*-mediated cell death in colon cancer.

Perhaps the most mechanistically novel finding of the present study is the delineation of an M2-TAM–*STAT3*–*RBMS3* regulatory axis in colon cancer. Cell–cell communication analysis from scRNA-seq data identified preferential signaling interactions between M2-TAMs and *RBMS3*-negative tumor cells, implicating M2-TAM-derived paracrine signals as suppressors of *RBMS3* expression in the TME. This was experimentally validated by demonstrating that co-culture with THP-1-derived M2-like macrophages significantly reduced *RBMS3* protein expression in HCT116 cells, accompanied by increased *P*-*STAT3* (Tyr705) levels in tumor cells—consistent with activation of the JAK-STAT signaling axis downstream of M2-derived paracrine cytokines (such as IL-6 and IL-10) identified in the CellChat analysis. Crucially, treatment with the selective *STAT3* inhibitor Stattic restored *RBMS3* expression in the context of M2-TAM co-culture, establishing a causal mechanistic link. The *STAT3* transcription factor is a well-established mediator of M2-TAM-driven immunosuppression in CRC. *STAT3* activation in COADtumor cells modulates the recruitment of Treg cells and M2-TAMs, reinforces immunosuppressive cytokine production, and promotes tumor progression. Targeting *STAT3* with Stattic in COADmouse models has been shown to markedly reduce the proportions of both TAMs and Tregs and to enhance the anti-tumor activity of CD8^+^ T cells, underscoring the therapeutic potential of *STAT3* blockade in CRC. The mechanism through which M2-like macrophages activate *STAT3* in tumor cells most likely involves paracrine cytokines—including IL-6, IL-10, and OSM—identified as top-ranked signals in the CellChat differential communication analysis. These cytokines are well-established extracellular activators of the JAK-STAT pathway, and their binding to cognate receptors on tumor cells triggers *STAT3* phosphorylation at Tyr705, which in turn may transcriptionally suppress *RBMS3* expression. How activated *STAT3* in turn suppresses *RBMS3* transcription remains to be fully elucidated; *STAT3* is known to bind promoter regions of tumor suppressor genes and to recruit epigenetic repressors including DNMT3A and EZH2 to silence their expression. Whether *STAT3* directly binds the *RBMS3* promoter or operates through intermediate transcriptional repressors or epigenetic mechanisms remains to be determined and represents an important direction for future investigation. The interconnection between M2-TAM-mediated *STAT3* activation, *RBMS3* suppression, and ferroptosis resistance identified in the present study is particularly relevant given emerging evidence linking tumor metabolic reprogramming with ferroptosis evasion in the immunosuppressive TME. Lactic acid, a major metabolite produced by glycolytic tumor cells, has recently been shown to promote M2 macrophage polarization and suppress GPX4-mediated ferroptosis in colon cancer cells, thereby facilitating tumor immune escape (Wang X. et al., 2026). Our data are consistent with the concept that THP-1-derived M2-like macrophages may suppress a pro-ferroptotic RNA-binding protein (*RBMS3*) *via STAT3* activation, potentially creating a positive feedback loop in which M2-like macrophages not only dampen anti-tumor immunity but also may disable the ferroptosis program in tumor cells in which M2-TAMs not only dampen anti-tumor immunity but also directly disable the ferroptosis program in tumor cells. This multi-layered suppression of ferroptosis by the immunosuppressive TME may represent a key mechanism underlying therapy resistance in advanced CRC. The M2-TAM–*STAT3*–*RBMS3*–ferroptosis axis also presents translational opportunities for combinatorial therapeutic strategies. *STAT3* inhibitors such as Stattic and napabucasin (BBI608) are currently under clinical evaluation in CRC, and our data suggest that *STAT3* blockade may simultaneously restore *RBMS3*-mediated ferroptosis sensitivity in tumor cells, offering a mechanistic basis for combining *STAT3*-targeted agents with ferroptosis inducers (e.g., RSL3, erastin analogs) or immune checkpoint inhibitors. Furthermore, M2-TAM reprogramming strategies—including CSF1R inhibitors and PPAR-γ agonists—may indirectly upregulate *RBMS3* by relieving *STAT3*-dependent suppression. Future preclinical studies in syngeneic CRC models should test whether *STAT3* inhibition combined with anti-PD-1/PD-L1 therapy or ferroptosis inducers yields synergistic anti-tumor effects through the *RBMS3* axis.

Several limitations merit consideration. First, the retrospective transcriptomic analyses preclude causal inferences, and prospective validation with protein-level *RBMS3* data in independent cohorts is warranted. Second, THP-1-derived M2-like macrophages represent a simplified *in vitro* model that may not fully recapitulate primary TAM biology, and the identified regulatory axis requires confirmation in syngeneic or humanized mouse models. Third, all functional experiments were performed in a single cell line (HCT116) with overexpression models only; siRNA-mediated knockdown and multi-cell-line validation will be addressed in future work. Fourth, ferroptosis-specific rescue experiments (e.g., ferrostatin-1) and direct lipid peroxidation assays (e.g., C11-BODIPY, MDA) were not performed, and are needed to formally confirm ferroptosis as the primary death modality. Finally, whether *RBMS3* directly binds GPX4 or *ACSL4* mRNA and whether *STAT3* suppresses *RBMS3* transcriptionally or epigenetically remain unresolved, and will require RIP-qPCR, mRNA stability assays, and promoter binding studies to fully define the M2-TAM–*STAT3*–*RBMS3*–ferroptosis axis at the molecular level. Additionally, Figure 4F in the current study measures only *RBMS3* protein expression following Stattic treatment; simultaneous detection of total *STAT3* and phospho-*STAT3* (Tyr705) to confirm effective *STAT3* inhibition under these experimental conditions will be included in future validation experiments.

Despite these limitations, the present study provides a comprehensive multi-dimensional analysis of *RBMS3* in colon cancer, establishing its pan-cancer prognostic relevance, clinical correlations, pro-ferroptotic function, and regulation by the immunosuppressive TME through a novel M2-TAM–*STAT3*–*RBMS3* axis. These findings nominate *RBMS3* as a clinically relevant biomarker and potential therapeutic target in colon cancer, and suggest that strategies aimed at restoring *RBMS3* expression—for example, through *STAT3* inhibition or combination approaches targeting M2-TAM reprogramming—may sensitize colon cancer cells to ferroptosis-based therapies, offering a promising avenue for future translational investigation. In conclusion, *RBMS3* is a prognostically relevant biomarker in COAD, associated with clinicopathological progression and ferroptosis-related tumor-cell states. M2 macrophages suppress *RBMS3* through *STAT3* activation, highlighting an immune–tumor regulatory axis with potential translational significance in COAD.

## Data Availability

The original contributions presented in the study are included in the article/supplementary material, further inquiries can be directed to the corresponding author.
